# Epigenetic Regulation of CXC Chemokine Expression by Environmental Electrophiles Through DNA Methyltransferase Inhibition

**DOI:** 10.3390/ijms252111592

**Published:** 2024-10-29

**Authors:** Tomoki Tsuchida, Sho Kubota, Shizuki Kamiuezono, Nobumasa Takasugi, Akihiro Ito, Yoshito Kumagai, Takashi Uehara

**Affiliations:** 1 Department of Medicinal Pharmacology, Graduate School of Medicine, Dentistry and Pharmaceutical Sciences, Okayama University, Okayama 700-8530, Japan; p77b19n9@s.okayama-u.ac.jp (T.T.); sho-kubota@okayama-u.ac.jp (S.K.); ntakasu@okayama-u.ac.jp (N.T.); 2 Department of Medicinal Pharmacology, Faculty of Pharmaceutical Sciences, Okayama University, Okayama 700-8530, Japan; paie95d9@s.okayama-u.ac.jp; 3 School of Life Sciences, Tokyo University of Pharmacy and Life Sciences, Tokyo 192-0392, Japan; aito@toyaku.ac.jp; 4 Graduate School of Pharmaceutical Sciences, Kyushu University, Fukuoka 812-8582, Japan; kumagai.yoshito.864@m.kyushu-u.ac.jp

**Keywords:** DNA methylation, DNA methyltransferase, chemical modification, chemokine, cell proliferation, toxicology, exposome, environmental electrophiles

## Abstract

Ubiquitously distributed environmental electrophiles covalently modify DNA and proteins, potentially leading to adverse health effects. However, the impacts of specific electrophiles on target proteins and their physiological roles remain largely unknown. In the present study, we focused on DNA methylation, which regulates gene expression and physiological responses. A total of 45 environmental electrophiles were screened for inhibitory effects on the activity of DNA methyltransferase 3B (DNMT3B), a key enzyme in DNA methylation, and four compounds were identified. We focused on 1,2-naphthoquinone (1,2-NQ), an air pollutant whose toxicity has been reported previously. Interestingly, we found that 1,2-NQ modified multiple lysine and histidine residues in DNMT3B, one of which was near the active site in DNMT3B. It was found that 1,2-NQ altered gene expression and evoked inflammatory responses in lung adenocarcinoma cell lines. Furthermore, we found that 1,2-NQ upregulated *CXCL8* expression through DNA demethylation of the distal enhancer and promoted cancer cell growth. Our study reveals novel mechanisms of epigenetic regulation by environmental electrophiles through the inhibition of DNMT3B activity and suggests their physiological impact.

## 1. Introduction

Humans are exposed to various environmental factors, such as factors in the natural environment, lifestyle, and diet, on a daily basis, which can affect well-being [1]. The concept of the “exposome” has been proposed to facilitate the understanding of the importance of environmental factors affecting human health and their potential impact [2]. However, because the exposome consists of all environmental exposures from conception to death, the number of factors to be measured in the entire exposome is too large to study. Therefore, factors are classified into three categories—specific external factors, general external factors, and internal factors—and the study of complete exposomes has been replaced by the study of partial exposomes [3]. Since then, many studies have focused on elucidating lifetime exposures to individual environmental factors [4,5]; however, the mechanistic details of the effects of these factors remain limited.

Environmental electrophiles have one or more electron-poor structures, such as α,β-unsaturated carbonyls and aldehydes, and easily react with biological nucleophiles such as DNA and proteins [6,7,8]. Because of these characteristics, environmental electrophiles have been recognized as the top priority components for the exposome study [9]. For example, 1,2-naphthoquinone (1,2-NQ) and its isomer 1,4-naphthoquinone (1,4-NQ) are present in ambient air and PM2.5, methylmercury is present in large fish such as tuna, cadmium is present in rice, methyl vinyl ketone (MVK) and crotonaldehyde are present in tobacco smoke, acrylamide is present in cooked food, and (E)-2-alkenals are present in vegetables [6,10]. Electrophile-protein modification leads to abnormal protein function and consequently disrupts physiological responses. For example, we previously reported that MVK modifies phosphatidylinositol-3 kinase (PI3K) and suppresses PI3K-Akt signaling, leading to altered physiological functions [10]. This evidence emphasizes that covalent modification by electrophiles can adversely affect physiological responses.

Epigenetic regulatory systems have recently attracted attention as regulatory mechanisms that alter physiological functions in response to environmental factors. DNA methylation plays a central role in mammalian cell differentiation and development and is closely associated with various physiological phenomena, such as gene expression regulation, genomic imprinting, and chromosome stabilization [11,12]. In mammals, DNA methyltransferases (DNMTs), including DNMT1, DNMT3A, and DNMT3B, play important roles in the maintenance and establishment of DNA methylation [13,14]. Deficiency of DNMT3B activity strongly contributes to pathogenesis and disease progression in immunodeficiency–centromeric instability–facial anomalies syndrome (ICF) through the disruption of gene expression [15]. These studies emphasize the important role of DNMTs in living organisms. Recent studies have reported that various environmental chemicals, such as nicotine in cigarette smoke [16], bisphenol A in polycarbonate plastics [17], and lead in Pb-based pipes and paints [18,19], disrupt DNA methylation. However, how environmental factors regulate the enzymatic activity of DNMTs remains unclear. Previously, we reported that nitric oxide, an endogenous electrophile-like mediator, covalently binds to DNMT3B and inhibits its enzymatic activity, causing epigenetic disorders and tumorigenesis [20]. Therefore, we hypothesized that certain environmental (xenobiotic) electrophiles affect the enzymatic activity of DNMTs and alter physiological responses.

In the present study, we aimed to identify environmental electrophiles that affect DNA methylation and elucidate their mechanisms and physiological effects. We identified four candidate environmental electrophiles that inhibit DNMT3B activity. Interestingly, 1,2-NQ is covalently bound to a lysine residue of DNMT3B that is very close to the active site. We found that 1,2-NQ induced upregulation of inflammatory cytokines, especially *CXCL8*, and DNA demethylation of the CXCL8 enhancer region. We investigated the relationships between *CXCL8* expression, lung cancer progression, and smoking history using The Cancer Genome Atlas (TCGA) database. Our study highlights the environmental electrophiles that regulate DNMT3B enzymatic activity through covalent modification and suggests their pathophysiological roles.

## 2. Results

### 2.1. 1,2-NQ Covalently Modifies DNMT3B and Inhibits Its Enzyme Activity

**Figure 1:**
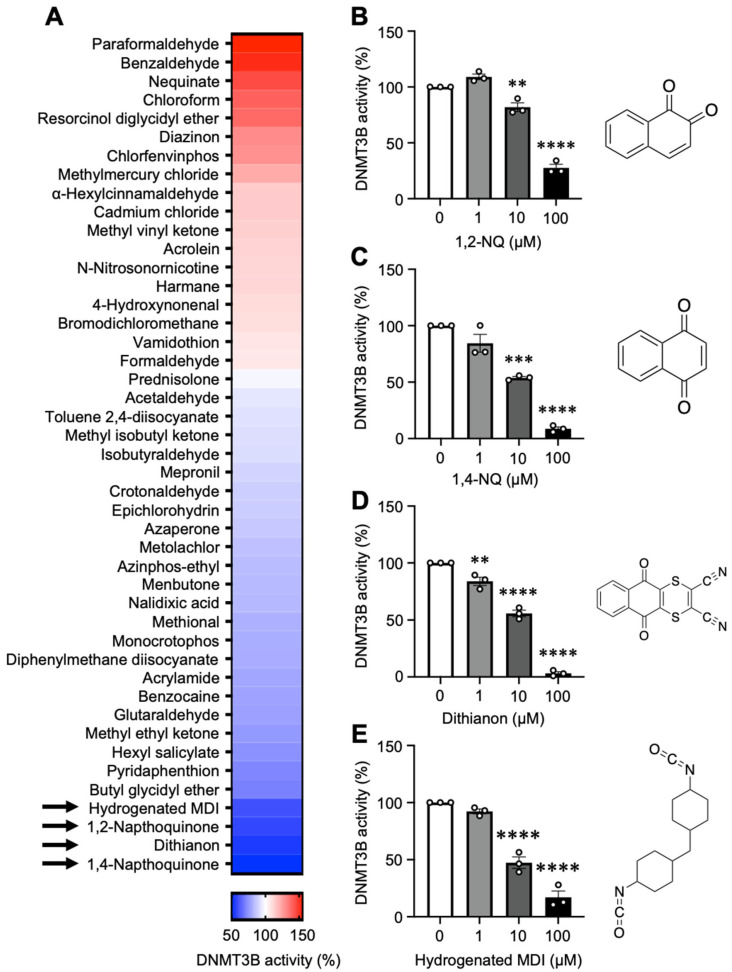
4 Electrophiles inhibit DNMT3B activity by an in vitro DNMT3B activity assay. (**A**) Heatmap showing the effects of 45 electrophiles (20 μM) on DNMT3B activity. The arrows indicate the four electrophiles identified to inhibit DNMT3B activity significantly. (**B**–**E**) Effects of the indicated concentrations of (**B**) 1,2-NQ, (**C**) 1,4-NQ, (**D**) dithianon, and (**E**) hydrogenated MDI on DNMT3B activity. The data are expressed as the means ± SEMs. *n* = 3, ** *p* < 0.01, *** *p* < 0.001, **** *p* < 0.0001 vs. 0 μM (**A**) 1,2-NQ, (**B**) 1,4-NQ, (**C**) dithianon, or (**D**) hydrogenated MDI. Statistical analyses were performed via one-way analysis of variance (ANOVA) with Dunnett’s multiple comparison test. 1,2-NQ; 1,2-naphthoquinone, 1,4-NQ; 1,4-naphthoquinone, MDI; methylene diphenyl diisocyanate.

To investigate the effects of different electrophiles on DNA methylation, we measured the in vitro activity of recombinant DNMT3B in the presence of electrophiles. A total of 45 electrophiles were selected (Table S1) according to the following criteria: (1) high abundance in the environment; (2) present in various products, such as air pollutants, pesticides, and medicines; (3) diverse structures, such as aldehydes, quinones, and α,β-unsaturated carbonyl compounds; (4) molecular weight less than 500; and (5) commercially available in Japan. We found that four electrophiles, 1,2-NQ, 1,4-NQ, dithianone, and hydrogenated methylene diphenyl diisocyanate (MDI), significantly attenuated DNMT3B activity (Figure 1A). Moreover, the inhibitory effects of these electrophiles were concentration-dependent (Figure 1B–E). Among these, we focused on 1,2-NQ due to multiple toxicities and its abundance in cigarette smoke and diesel exhaust and because it has been reported to have various toxicities associated with protein adduct formation [21]. DNA methyltransferases include DNMT1, which is involved in the maintenance of DNA methylation [22], and DNMT3A and DNMT3B, which are involved in de novo DNA methylation [23]; thus, we examined the inhibitory effects of 1,2-NQ for each DNMT subtype. The findings showed that 1,2-NQ reduced the catalytic activity of all the DNMTs in a concentration-dependent manner (Figure S1A–C). These results suggest that 1,2-NQ can act as an epigenetic regulator via the inhibition of DNMT activity.

**Figure 2:**
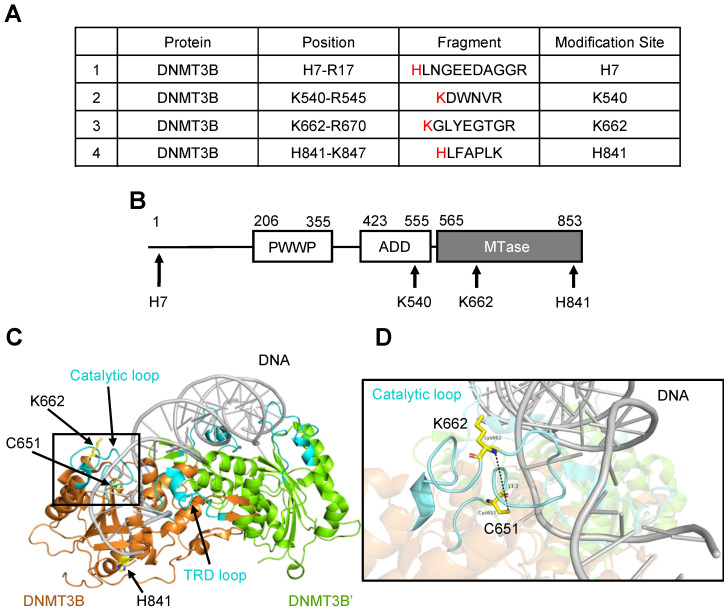
1,2-NQ covalently modified DNMT3B by LC–MS/MS analysis using recombinant DNMT3B protein. (**A**) Identification of 1,2-NQ-binding sites in recombinant DNMT3B/DNMT3L via LC–MS/MS. The recombinant DNMT3B/DNMT3L protein (1 µg) was incubated with 20 µM 1,2-NQ for 30 min at RT. 1,2-NQ modification sites are shown in red. (**B**) Domain structure of DNMT3B. DNMT3B comprises three domains: PWWP (Pro-Trp-Trp-Pro), ADD (ATRX-DNMT3-DNMT3L), and methyltransferase (MTase). The arrows show the 1,2-NQ modification sites in DNMT3B. (**C**) Structures of the DNMT3B MTase domain (residues 571–853) bound to DNA were reconstructed using PyMOL (PDB 6u8p) [29]. The catalytic loop (residues 648–672) and TRD loop (residues 772–791) are shown in cyan. The catalytic cysteine C651 and 1,2-NQ modification sites K662 and H841 are shown in yellow. (**D**) Enlarged view of DNA interactions in the catalytic loop. The DNA is shown in gray, and the catalytic loop is shown in cyan. The distance between the nitrogen atom of K662 and the sulfur atom of C651 was 11.2 Å. TRD; Target recognition domain.

1,2-NQ with an α,β-unsaturated carbonyl and quinone structures is reactive with protein nucleophiles, such as cysteine thiolate (Cys), lysine amine (Lys), and histidine imidazole (His), and can form covalent modifications [24,25,26,27]. Therefore, we investigated whether modification by 1,2-NQ plays an important role in the loss of function of DNMT3B. We incubated recombinant DNMT3B and 1,2-NQ and performed liquid chromatography–tandem mass spectrometry (LC–MS/MS). LC–MS/MS showed that 1,2-NQ covalently bound to His7, Lys540, Lys662, and His841 within DNMT3B (Figure 2A and Figure S2). Of these residues, His7 is located in the N-terminal variable region; Lys540 is in the ADD domain, which interacts with the methyltransferase (MTase) domain related to autoinhibition [28]; and Lys662 and His841 are in the MTase domain (Figure 2B). We checked the conformational structure of the DNMT3B MTase domain using PyMOL (version 2.5.2) to expect the effects of the covalently bound 1,2-NQ [29] (Figure 2C). Interestingly, we found that Lys662 is in a catalytic loop that interacts with DNA and is situated near Cys651, which plays an important role in DNA methylation (Figure 2D). Thus, our data indicate that 1,2-NQ covalently modifies DNMT3B and that covalent modifications of DNMT3B may block DNMT3B-DNA interactions, leading to abrogated enzymatic activity.

### 2.2. 1,2-NQ Exposure Upregulates CXC Chemokines and Activates Inflammatory Responses

**Figure 3:**
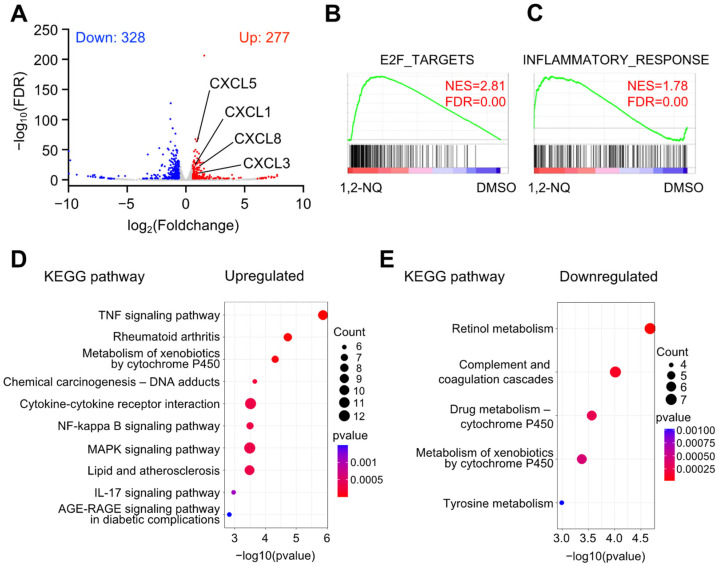
1,2-NQ-induced genes are related to inflammatory responses in A549 cells. (**A**) Volcano plot showing the differentially expressed genes (DEGs) between 1,2-NQ-treated and dimethyl sulfoxide (DMSO)-treated (control) A549 cells. The upregulated genes are shown in red (FDR < 0.01, log_2_-fold change > 0.58), the downregulated genes are shown in blue (FDR < 0.01, log_2_-fold change < −0.58), and unaffected genes are shown in gray. (**B**,**C**) Gene set enrichment analysis (GSEA) of Hallmark gene sets in A549 cells after 1,2-NQ treatment versus control DMSO. Enrichment plots for (**B**) HALLMARK_E2F_TARGETS and (**C**) HALLMARK_INFLAMMATORY_RESPONSE. (**D**,**E**) Dot plots showing the results of KEGG pathway enrichment analysis performed for (**D**) upregulated genes and (**E**) downregulated genes in A549 cells after 1,2-NQ treatment versus the DMSO control. NES; normalized enrichment score, FDR; false discovery rate, 1,2-NQ; 1,2-naphthoquinone.

To investigate whether the air pollutant 1,2-NQ is an epigenetic factor affecting cellular homeostasis, we performed RNA sequencing (RNA–seq) in A549 cells exposed to 1,2-NQ for 72 h. First, we identified the differentially expressed genes (DEGs) between the 1,2-NQ treated and control groups. We found that 1,2-NQ treatment upregulated 277 genes (Up-DEGs) and downregulated 328 genes (Down-DEGs) (Figure 3A). Gene set enrichment analysis (GSEA) revealed that 1,2-NQ activated cell cycle regulators such as E2F target genes and inflammatory response genes (Figure 3B,C). Kyoto Encyclopedia of Genes and Genomes (KEGG) pathway enrichment analysis revealed that Up-DEGs were involved in inflammation, including the TNF, NF-κB, and IL-17 signaling pathways (Figure 3D), whereas Down-DEGs were involved in metabolism (Figure 3E). These results suggest that 1,2-NQ activates various signaling pathways related to inflammatory responses.

**Figure 4:**
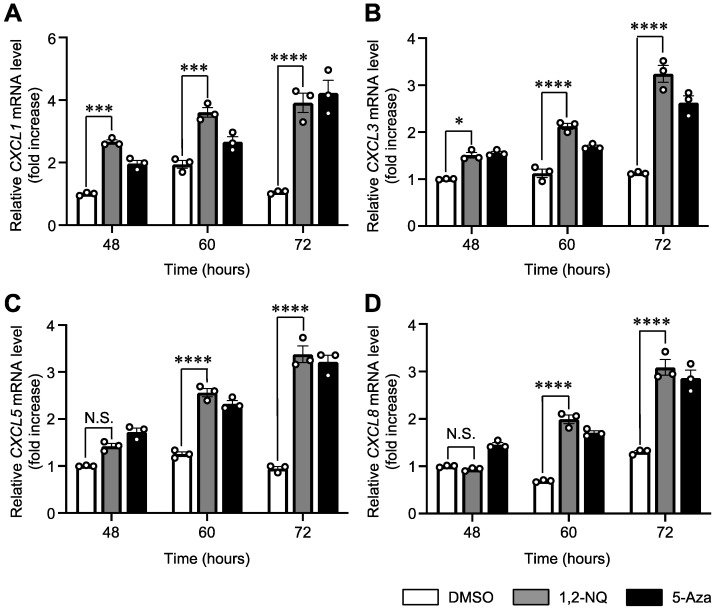
Treatment with 1,2-NQ or the DNMT inhibitor 5-aza upregulated the expression of CXC chemokines in A549 cells. (**A**–**D**) A549 cells were exposed to 10 μM 1,2-NQ or 10 μM 5-aza for the indicated times, and total RNA was extracted. RT–qPCR analysis of (**A**) *CXCL1*, (**B**) *CXCL3*, (**C**) *CXCL5*, and (**D**) *CXCL8* expression. Control DMSO is shown in white, 1,2-NQ is shown in gray, and 5-aza is shown in black. The data are expressed as the means ± SEMs. *n* = 3, N.S.: not significant, * *p* < 0.05, *** *p* < 0.001, **** *p* < 0.0001 versus control DMSO at the same timepoint. Statistical analyses were performed via one-way ANOVA with Tukey’s multiple comparison test. 1,2-NQ; 1,2-naphthoquinone, 5-Aza; 5-aza-2′-deoxycytidine.

As target genes of 1,2-NQ-induced epigenetic changes, we focused on CXC chemokines, which play important roles in the regulation of inflammation [30,31]. We performed RT–qPCR to detect the changes over time in the gene expression of *CXCL1*, *CXCL3*, *CXCL5*, and *CXCL8*, which were identified as Up-DEGs via RNA–seq analysis (Figure 3A). Consequently, 1,2-NQ exposure for more than 48 h increased CXC chemokine mRNA levels in a time-dependent manner (Figure 4A–D). However, exposure to 1,2-NQ for 24 h did not induce the expression of CXC family genes (Figure S3A–D). In addition, 5-aza-2′-deoxycitidine (5-aza), a DNMT inhibitor, upregulated CXC chemokine expression in a time-dependent manner, as well as 1,2-NQ (Figure 4A–D). Previous studies have shown that TNF-α rapidly stimulates *CXCL1* and *CXCL8* production via NF-κB signaling [32,33]. Therefore, we analyzed the effects of TNF-α on CXC chemokine expression. We found that TNF-α induced CXC chemokine expression more rapidly than did 1,2-NQ or 5-aza (Figure S3E–H). These data suggest that 1,2-NQ, unlike TNF-α, induces CXC chemokine expression by regulating DNA methylation.

### 2.3. 1,2-NQ Induces DNA Hypomethylation and Promotes p65 Recruitment to the CXCL8 Enhancer Region

**Figure 5:**
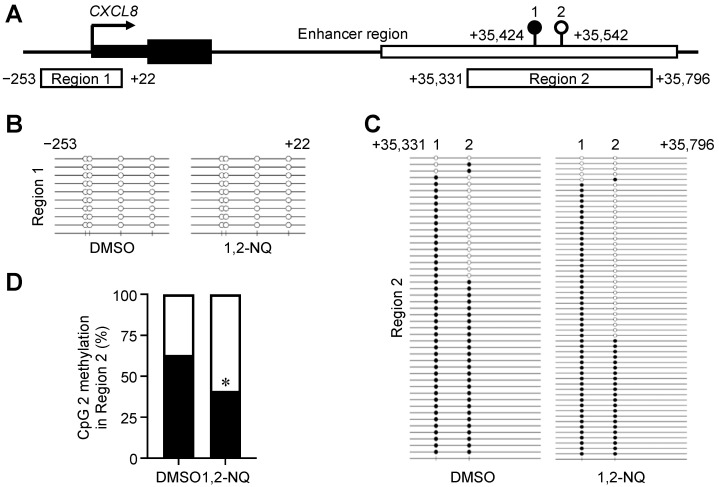
1,2-NQ induces demethylation of the CXCL8 enhancer region in A549 cells. (**A**) Schematic diagram of the bisulfite target regions. Two different bisulfite targets, −253 to +22 (designated Region 1 within the promoter region) and +35,331 to +35,796 (designated Region 2 within the enhancer region) are indicated. (**B**,**C**) A549 cells were exposed to 10 μM 1,2-NQ for 72 h, and DNA was extracted for bisulfite sequencing. Methylation levels in (**B**) Region 1 and (**C**) Region 2. The open circles represent demethylated CpG sites, and the closed circles represent methylated CpG sites. (**D**) Methylation levels at the upper cytosine in Region 2 (+35,542). The open bars represent demethylated CpG sites, and the closed bars represent methylated CpG sites. *n* = 46 (DMSO), *n* = 56 (1,2-NQ), * *p* < 0.05, versus control DMSO. Statistical analyses were performed using a two-tailed Fisher’s exact test.

To examine whether the epigenetic changes induced by 1,2-NQ stimulate the expression of CXC chemokines, we performed bisulfite sequencing to elucidate the methylation levels in specific regions (Figure 5A). Since previous reports have shown that *CXCL8* (IL-8) mRNA levels are negatively correlated with DNA methylation levels in the promoter region [34], we focused on *CXCL8* among the CXC chemokines. Bisulfite sequencing revealed that the CXCL8 promoter region was completely demethylated in both the control groups and the 1,2-NQ-treated groups (Figure 5B). Next, we designated and analyzed the downstream CXCL8 enhancer region, which contains the peaks of H3K4me1, H3K27ac, and p65 under TNF-α stimulation (Figure S4). We found that 1,2-NQ treatment decreased the methylation level at the upstream CpG site (CpG 2) in the CXCL8 enhancer region (Figure 5C,D).

**Figure 6:**
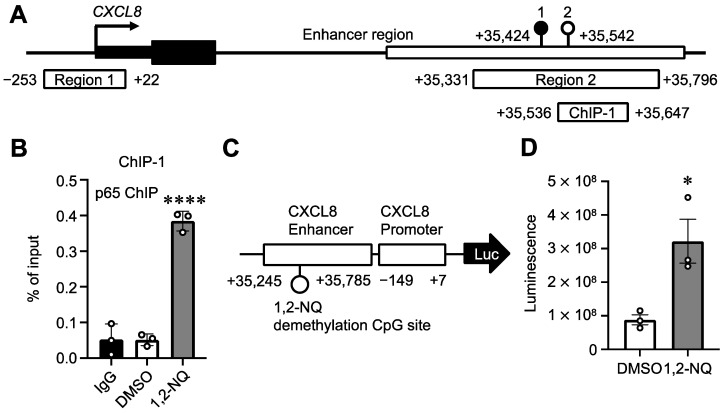
1,2-NQ promotes p65 recruitment and activates CXCL8 enhancer activity in A549 cells. (**A**) Schematic diagram of a ChIP target region near CXCL8. The ChIP target region, +35,536 to +35,647 (designated ChIP-1 within the enhancer region) is indicated. (**B**) Chromatin from A549 cells exposed to 10 μM 1,2-NQ for 72 h was precipitated with a p65 antibody or control IgG. Binding of p65 to the ChIP-1 region. The data are expressed as the means ± SEMs. *n* = 3, **** *p* < 0.0001 versus control DMSO. Statistical analyses were performed via one-way ANOVA with Dunnett’s multiple comparison test. (**C**) Schematic illustration of the used luciferase reporter. (**D**) HEK293T cells were transiently transfected with the luciferase reporter and DNMT3B and exposed to 10 μM 1,2-NQ for 72 h, followed by the luciferase reporter assay. The data are expressed as the means ± SEMs. *n* = 3, * *p* < 0.05 versus control DMSO. Statistical analyses were performed using unpaired two-tailed Student’s *t* tests. 1,2-NQ; 1,2-naphthoquinone.

*CXCL8* production in A549 cells is dependent on NF-κB and MEK/ERK MAP kinases [35,36]. Therefore, we performed chromatin immunoprecipitation qPCR (ChIP–qPCR) to detect the binding of NF-κB p65 to the demethylated enhancer region. We showed that 1,2-NQ treatment increased p65 binding to the ChIP-1 region, including the 1,2-NQ-demethylated CpG site, within the CXCL8 enhancer region (Figure 6A,B). In contrast, 1,2-NQ treatment did not induce transient and apparent p65 nuclear translocation, as detected by TNF-α treatment (Figure S5). Next, we constructed a luciferase reporter containing the CXCL8 enhancer region to measure the enhancer activity (Figure 6C). We performed a luciferase reporter assay by co-transfection of DNMT3B with the luciferase reporter into cells and found that 1,2-NQ increased the luciferase activity (Figure 6D). These results indicate that 1,2-NQ treatment increased *CXCL8* mRNA levels through enhancer demethylation, activation, and p65 recruitment.

### 2.4. 1,2-NQ Stimulates Aberrant Cancer Cell Proliferation via CXCL8 Autocrine Signaling

**Figure 7:**
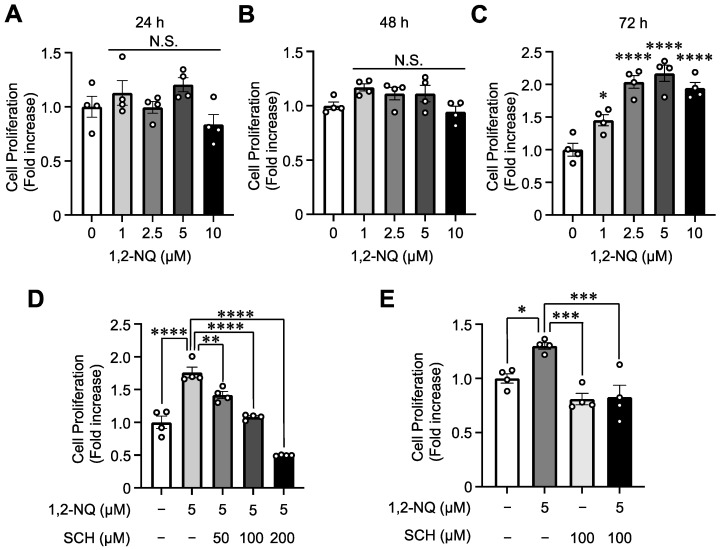
1,2-NQ promotes cell proliferation via autocrine CXCL8 signaling in A549 cells. (**A**–**E**) Cell proliferation was detected using the WST-8 assay. (**A**–**C**) A549 cells were incubated in serum-free medium for 24 h and then exposed to the indicated concentrations of 1,2-NQ for (**A**) 24, (**B**) 48, or (**C**) 72 h. (**D**) A549 cells were incubated in serum-free medium for 20 h and then exposed to the indicated concentrations of SCH-527123 (a CXCR 1/2 antagonist) for 4 h. The cells were treated with 5 μM 1,2-NQ for 72 h. (**E**) A549 cells were incubated in serum-free medium for 20 h and treated with 100 μM SCH-527123 or DMSO for 4 h. The cells were treated with 5 μM 1,2-NQ for 72 h. The data are expressed as the means ± SEMs. *n* = 3, N.S.: not significant, * *p* < 0.05, ** *p* < 0.01, *** *p* < 0.001, **** *p* < 0.0001 versus (**A**–**C**) 0 μM 1,2-NQ or (**D**,**E**) 5 μM 1,2-NQ alone. Statistical analyses were performed via one-way ANOVA with Dunnett’s multiple comparison test. 1,2-NQ; 1,2-naphthoquinone, SCH; SCH-527123.

Previous studies have reported that CXCL8 promotes cell proliferation in multiple cancers via the CXCL8–CXCR1/2 axis [33]. Therefore, we investigated whether 1,2-NQ-induced CXCL8 expression promotes lung cancer cell growth. We performed a WST-8 assay in serum-free medium to exclude the effects of other growth factors present in serum. Exposure to 1,2-NQ for 72 h, but not 24 or 48 h, significantly promoted cell growth (Figure 7A–C). To determine the contribution of the CXCL8–CXCR1/2 axis to cell growth, we used SCH-527123 (a CXCR1/2 antagonist). Pretreatment with SCH-527123 diminished 1,2-NQ-induced cell proliferation in a concentration-dependent manner. However, SCH-527123 alone did not affect cell growth (Figure 7D,E). These results demonstrated that 1,2-NQ epigenetically induces *CXCL8* expression, leading to aberrant cell growth via the CXCL8–CXCR1/2 axis.

### 2.5. High CXCL8 Expression Is Associated with Lung Cancer Prognosis and Smoking History in TCGA Database

**Figure 8:**
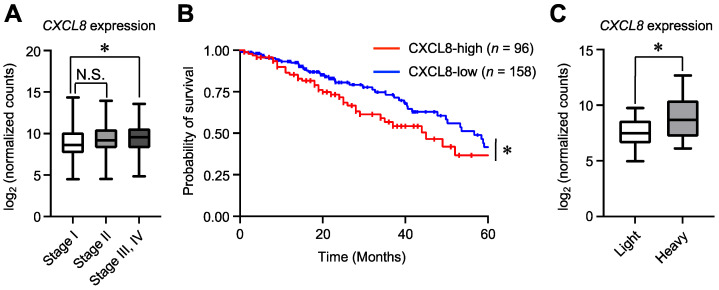
*CXCL8* expression is positively correlated with poor prognosis and cumulative smoking history in patients with lung cancer by a computational analysis using TCGA database. (**A**) The expression of *CXCL8* in patients with LUAD of different tumor stages. The data are presented as box plots. The centerlines indicate the median, and the box limits indicate the 25th and 75th percentiles, respectively. Stage I (*n* = 275), Stage II (*n* = 122), Stage III and IV (*n* = 110), N.S.: not significant, * *p* < 0.05, versus the Stage I group. Statistical analyses were performed via one-way ANOVA with Tukey’s multiple comparison test. (**B**) Kaplan–Meier plots comparing the overall survival of patients with low *CXCL8* (*n* = 158) and high *CXCL8* (*n* = 96) expression levels at the mRNA level. * *p* < 0.05. Statistical analyses were performed using log-rank tests. (**C**) The expression of *CXCL8* in light (light) and heavy (heavy) smokers. Among cases in the TCGA database, those in the top 20% of smoking pack-years were classified as heavy smokers, and those in the bottom 20% were classified as light smokers. The data are presented as box plots. Light and heavy smokers (*n* = 70); * *p* < 0.05. Statistical analyses were performed using unpaired two-tailed Student’s *t* tests.

Finally, we reanalyzed data from the TCGA database [37] to investigate whether *CXCL8* expression was related to cancer progression. We found that CXCL8 expression was positively correlated with lung cancer stage (Figure 8A). Interestingly, Kaplan–Meier survival analysis indicated that high *CXCL8* expression was significantly associated with shorter overall survival (Figure 8B). Because 1,2-NQ is a representative metabolite of naphthalene, which is abundant in cigarette smoke [38], we hypothesized that patients with a long history of smoking have higher *CXCL8* expression due to the exposure of 1,2-NQ. Thus, we analyzed the relationship between *CXCL8* expression and smoking history in pack-years. Surprisingly, we found that *CXCL8* expression was higher in heavy smokers (top 20th percentile) than in light smokers (bottom 20th percentile) (Figure 8C). Taken together, our data showed that cigarette smoking, along with 1,2-NQ exposure, may aggravate lung cancer by promoting *CXCL8* expression.

## 3. Discussion

Recently, it has been recognized that environmental factors such as environmental chemicals and dietary nutrients are deeply involved in the epigenetic regulation of genes. In this study, we found that 1,2-NQ, an environmental electrophile, alters epigenetic regulation by modifying DNMT3B, leading to aberrant cell growth in a lung cancer cell line. Our study provides novel mechanisms for the use of 1,2-NQ, an environmental electrophile involved in epigenetic regulation via covalent modifications.

We identified four electrophiles that inhibited DNMT3B activity in a dose-dependent manner (Figure 1). Interestingly, three of these four electrophiles exhibit naphthoquinone structures (Table S1). This class of electrophiles exhibits high reactivity with proteins [39], suggesting that high electrophilicity plays a key role in DNMT3B inhibition. Among these naphthoquinones, we focused on 1,2-NQ, which is present in air pollutants such as diesel exhaust, tobacco smoke, and PM2.5 [40,41,42]. 1,2-NQ was covalently bound to lysine and histidine residues in DNMT3B (Figure 2A). Although further studies are needed to determine whether these residues contribute to DNMT3B inhibition, we found that the Lys662 in the MTase domain, a 1,2-NQ modification site, was very close to Cys651 (Figure 2C,D). Previous studies have shown that sulforaphane with the isothiocyanate structure, and nanaomycin A, with the anthraquinone structure, interact with residues near Cys651 and block the entry of S-adenosylmethionine, which is critical for methyltransferase activity [43,44]. In addition, we compared the amino acid sequences of the DNMT family members and found that 1,2-NQ modification sites in the MTase domain were conserved in DNMT3A but not in DNMT1 (Figure S6A–D). Although further studies are needed to elucidate the mechanism of DNMT1 inhibition by 1,2-NQ, our data suggest that DNMT3 inhibition may occur via adduct formation by 1,2-NQ.

To elucidate the effects of 1,2-NQ on cellular homeostasis, we conducted experiments in A549 lung cancer cells. Our RNA sequencing analyses suggested that 1,2-NQ induced an inflammatory response via the TNF or NF-κB signaling pathway (Figure 3). Chronic inflammation induces the expression of cytokines and chemokines that stimulate cell growth and survival, promote angiogenesis, and are closely associated with cancer progression [45]. Previous studies have reported that CXCL8–CXCR1/2 signaling is involved in inflammation, tumor enhancement, and immunotherapy resistance and plays an important role in cancer progression [46,47]. Consistent with this evidence, we showed that 1,2-NQ stimulated cell proliferation through CXCL8–CXCR1/2 signaling (Figure 7). Recent studies have reported that cigarette smoke and PM2.5, both of which contain 1,2-NQ, promote lung cancer progression [48,49]. We also showed that high *CXCL8* mRNA levels were positively correlated with lung cancer malignancy (Figure 8). Although further studies are needed to determine the contribution of 1,2-NQ to *CXCL8* expression, our data suggest that environmental electrophiles such as 1,2-NQ may contribute, at least in part, to the exacerbation of lung cancer.

Unlike TNF-α (Figure 4 and Figure S3), a known fast CXCL8 inducer, both 1,2-NQ and 5-aza, a known DNMT inhibitor, induced *CXCL8* gene expression gradually [32,33]. These results suggest that the mechanism by which 1,2-NQ regulates *CXCL8* expression, perhaps through DNA methylation, is likely distinct from that of TNF-α, which activates AP-1 and NF-κB. In support of this result, a previous report showed that DNA methylation of CXCL8 plays a key role in gene expression [34,50,51,52]. Although the CXCL8 promoter region was unmethylated in this cell line, we detected hypomethylation of a CpG site in the CXCL8 distal enhancer after 1,2-NQ treatment (Figure 5C,D). DNA methylation is generally known as a repressive epigenetic marker; however, its potential role as an enhancer is poorly understood [11,53]. Interestingly, a recent study demonstrated that DNA methylation of enhancers rather than promoters plays a critical role in tumorigenesis and immune infiltration in lung squamous cell carcinoma, which often occurs in smokers [54]. This evidence emphasizes that the DNA methylation of enhancers plays an important role in cancer development and progression. Moreover, a recent study reported that transcription factor occupancy in enhancers is dependent on DNA methylation [55]. In this study, we found that 1,2-NQ treatment increased the recruitment of NF-κB p65 without inducing its transient nuclear translocation (Figure 6B and Figure S5). We previously reported that 1,2-NQ suppressed lipopolysaccharide-induced NF-κB signaling by inhibiting IKKβ in RAW 264.7 macrophages [56]. According to this evidence, 1,2-NQ may induce CXCL8 expression independently of NF-κB signaling. These results suggest that 1,2-NQ-induced DNA demethylation alone may be involved in the recruitment of steady-state levels of p65 to CXCL8 enhancer regions. Moreover, we revealed that 1,2-NQ increased the CXCL8 enhancer activity (Figure 6C,D). Our data indicate that 1,2-NQ-induced enhancer demethylation is strongly associated with the epigenetic regulation of *CXCL8*.

We assumed that the exposure conditions for 1,2-NQ were consistent with the actual human exposure conditions, as shown in Figure 1, Figure 2, Figure 3, Figure 4, Figure 5, Figure 6 and Figure 7. For example, a previous study reported that the atmosphere in Birmingham, United Kingdom, contained 3374 pg/m^3^ 1,2-NQ [57]. If the daily respiratory volume is converted to 10 m^3^, the exposure is approximately 30 µg/day, which is approximately 0.2 µmol of 1,2-NQ per day. In this study, the maximum treatment volume for A549 cells was 10 µM in 10 mL of medium, which is equivalent to 0.1 µmol. This calculation indicates that the experimental volumes were not significantly different from those posed under normal conditions. Above all, 1,2-NQ is not the sole chemical substance present in the atmosphere, as it is constantly exposed to a variety of electrophiles on a daily basis [6,10]. Recent studies have shown that combined exposure to electrophiles in pesticides and air pollutants and heavy metals results in additive or synergistic toxicity [58,59]. Therefore, the aberrant DNA methylation mechanism revealed in our study may occur especially when individuals are exposed to numerous electrophiles, as is the case in daily life. Considering these findings, elucidation of the mechanisms and pathophysiological effects of combined exposure to electrophiles will lead to a fuller understanding of the adverse effects of these environmental exposures.

This study has some important limitations. First, the effects of covalent modifications by 1,2-NQ on DNMT3B enzymatic activity remain unclear. We reconstructed a three-dimensional structure and suggested a mechanism for the inhibition of DNMT3B enzymatic activity by 1,2-NQ, but further studies using DNMT3B mutants without 1,2-NQ modification sites are required. Furthermore, the contributions of 1,2-NQ to lung cancer progression have not been elucidated. Our study only showed that *CXCL8* expression, which is related to lung cancer prognosis, was increased by 1,2-NQ in vitro and correlated with smoking history in the TCGA database. Further studies using in vivo models are needed to clarify the roles of 1,2-NQ in lung cancer progression.

To our knowledge, this is the first study to present evidence that environmental electrophiles regulate DNA methylation by inhibiting DNMT enzymatic activity and promoting cell proliferation. Our findings reveal novel mechanisms by which environmental electrophiles promote cancer progression and may lead to the development of therapeutic strategies for various diseases driven by environmental factors.

## 4. Materials and Methods

### 4.1. Reagents and Antibodies

The electrophiles used are listed in Table S1. Antibodies against rabbit IgG (FUJIFILM Wako Pure Chemical Corporation, Osaka, Japan, #148-09551), NF-κB p65 (Cell Signaling Technology, Danvers, MA, USA, #D14E12), Lamin A/C (Proteintech, Rosemont, IL, USA, #10298-1-AP), and α-tubulin (FUJIFILM Wako Pure Chemical Corporation, #013-25033) were purchased from the indicated vendors. SCH-527123 (S8506) and TNF-α (HZ-1014) were purchased from Selleck Chemical (Houston, TX, USA) and Proteintech, respectively.

### 4.2. In Vitro DNMT Activity Assay

The in vitro DNMT activity assay was performed as described previously [20]. We used the DNMT Universal Chemiluminescent Assay Kit (BPS Biosciences, San Diego, CA, USA) to assess the inhibitory effects of 1,2-NQ on each DNMT isoform (DNMT1, DNMT3A, and DNMT3B) and the DNMT3B Chemiluminescent Assay Kit (BPS Biosciences) to screen 45 electrophiles for the ability to inhibit DNMT3B. Briefly, each recombinant DNMT isoform was incubated with a solvent or 20 μM electrophile, 40 μM S-adenosyl methionine, or assay buffer containing dithiothreitol in DNA substrate-coated 96-well plates for 2 h at 37 °C. After the reaction, the methylated DNA substrates were labeled with anti-5-methylcytosine antibody for 1 h at room temperature (RT). Finally, the samples were incubated with horse radish peroxidase (HRP)-conjugated secondary antibody for 30 min at RT, and HRP substrates were added. The chemiluminescence intensity was measured using the Glomax-Multi Detection System (Promega, Madison, WI, USA).

### 4.3. LC–MS/MS Analysis and Multiple Sequence Alignment

Complexed recombinant DNMT3B/DNMT3L (1 μg; Active Motif, Carlsbad, CA, USA) was incubated with 20 µM 1,2-NQ for 30 min at RT. Proteins were digested with trypsin (Promega) at 37 °C overnight and separated via high-performance liquid chromatography (HPLC) (EASY-nLC 1000) (Thermo Fisher Scientific, Waltham, MA, USA). HPLC was performed using a NANO-HPLC capillary C18 column (Nikkyo Technos, Tokyo, Japan) (0.075 × 150 mm) at 45 °C. Gradient elution was performed using two solvents (Solvent A, 0.1% formic acid; Solvent B, 0.1% formic acid mixed with 100% acetonitrile) at a flow rate of 300 nL/min. The trap column used was an Acclaim PepMap 100 precolumn (100 μm × 2 cm) (Thermo Fisher Scientific). MS and MS/MS scans were performed using a Q Exactive Hybrid Quadrupole-Orbitrap mass spectrometer with a positive-mode nanospray ion source (Thermo Fisher Scientific). The MS and MS/MS data were acquired using the data-dependent top10 method and were used to search the human SwissProt database with Proteome Discoverer (version 2.4, Thermo Fisher Scientific) with MASCOT search engine software (version 2.7.0, Matrix Science, Columbia, SC, USA). Each DNMT sequence was aligned with Clustal Omega software (https://www.ebi.ac.uk/jdispatcher/msa/clustalo, accessed on 26 August 2024) [60].

### 4.4. Three-Dimensional Structure of DNMT3B MTase Domain

Structures of the DNMT3B MTase domain (residues 571–853) bound to DNA were reconstructed using PyMOL (PDB 6u8p) [29].

### 4.5. Cell Culture

A549 human lung adenocarcinoma epithelial cell line was obtained from ATCC (CCL-185) and HEK293T human embryonic kidney cell line was obtained from ATCC (CRL-3216). A549 cells and HEK293T cells were cultured in Dulbecco’s modified Eagle medium (DMEM; FUJIFILM Wako Pure Chemical Corporation) supplemented with 10% (*v*/*v*) heat-inactivated fetal bovine serum (FBS, Belize City, Belize; Sigma-Aldrich, St. Louis, MO, USA) and 1% penicillin/streptomycin (FUJIFILM Wako Pure Chemical Corporation) at 37 °C in a humidified atmosphere containing 5% CO_2_/95% air.

### 4.6. RNA Sequencing and Data Analysis

A549 cells were treated with 10 μM 1,2-NQ for 72 h. Total RNA was extracted using the Maxwell RSC simply RNA Cell Kit (Promega). RNA quality control was performed using Tapestation 4200 High-Sensitivity RNA tapes (Agilent Technologies, Santa Clara, CA, USA). Poly(A) RNA extraction and fragmentation from total RNA (100 ng) were performed using the NEBNext Poly(A) mRNA Magnetic Isolation Module (New England BioLabs, Ipswich, MA, USA) and the NEBNext Ultra II RNA Library Prep Kit for Illumina (New England BioLabs). Next, cDNA synthesis was performed using the NEBNext Ultra II RNA Library Prep Kit for Illumina, followed by adapter tagging using the NEBNext Adaptor (New England BioLabs). The sequencing libraries were prepared by amplifying cDNA via PCR under the following conditions: 98 °C for 30 s, followed by 11 cycles of 98 °C for 10 s and 65 °C for 75 s. Sequencing of the 50 bp cDNA regions and index sequences was performed using the NovaSeq 6000 SP Reagent Kit V1.5 (Illumina, San Diego, CA, USA). All sequence data files were analyzed with default parameters using the CLC Genomics Workbench 22.0.1 (Qiagen, GmbH, Hilden, Germany). Quantitative analysis was performed using R software (4.2.3). Gene expression was normalized to transcripts per million (TPM) and upregulated or downregulated DEGs were identified using the edgeR (3.40.2) package [61,62,63,64]. Volcano plots were generated via GraphPad Prism 10 (GraphPad). Gene set enrichment analysis (GSEA) was performed using GSEA software (4.1.0). KEGG pathway enrichment analysis of the upregulated or downregulated DEGs was performed using the clusterProfiler (4.6.2) package [65,66], and the org.Hs.eg.db (3.16.0) package as a library.

### 4.7. RT–qPCR

RT–qPCR was performed as described previously [20]. Total RNA was extracted from A549 cells using TRI Reagent (Molecular Research Center, Cincinnati, OH, USA), and cDNA was synthesized from total RNA using ReverTra Ace RT qPCR Master Mix (TaKaRa, San Jose, CA, USA) according to the manufacturer’s protocol. qPCR was performed using KOD SYBR qPCR Mix (TOYOBO, Osaka, Japan) under the following conditions: 98 °C for 2 min, followed by 40 cycles of 98 °C for 10 s, 60 °C for 10 s, and 68 °C for 30 s. The primers used are listed in Table S2. Gene expression data were quantified via the 2^−∆∆Ct^ relative quantification method, using ACTB for normalization.

### 4.8. Targeted Bisulfite Sequencing

Genomic DNA was extracted from A549 cells using the Wizard Genomic DNA Purification Kit (Promega), and bisulfite conversion was performed using the Epitect Fast Bisulfite Conversion Kit (Qiagen) according to the manufacturer’s protocol. After bisulfite conversion, region 1 (within the CXCL8 promoter) and region 2 (within the CXCL8 enhancer) were amplified via PCR using EpiTaq HS (TaKaRa). The primers used are listed in Table S2. The PCR products were purified via electrophoresis on 2% agarose gels with 0.5 ng/mL ethidium bromide, and gel extraction was performed via the FavorPrep GEL/PCR Purification Mini Kit (FAVORGEN, Ping Tung, Taiwan). Next, the PCR products were cloned and inserted into a pMD20-T vector using a Mighty TA cloning kit (TaKaRa) and transformed into competent Escherichia coli DH5α cells. Insert-check PCR was performed using the primers listed in Table S2. Plasmids from the insert-positive colonies were extracted using the FavorPrep Plasmid Extraction Mini Kit (FAVORGEN) and sequenced. The methylation status of each plasmid was quantified using QUMA (http://quma.cdb.riken.jp/, accessed on 30 October 2023).

### 4.9. Chromatin Immunoprecipitation qPCR (ChIP–qPCR)

ChIP was performed as previously described [67]. Briefly, A549 cells were fixed for 5 min in 1.0% paraformaldehyde (FUJIFILM Wako Pure Chemical Corporation) diluted in PBS at 37 °C. Fixed cells were lysed in ice-cold radioimmunoprecipitation buffer (50 mM Tris-HCl (pH 8.0), 150 mM NaCl, 2 mM EDTA, 1% NP-40, 0.5% sodium deoxycholate, 0.1% SDS, and an EDTA-free protease inhibitor cocktail) and sonicated 15 times at an amplitude of 50% for 10 sec at 1 min intervals. The samples were incubated with an anti-IgG antibody or an anti-NF-κB p65 antibody conjugated with Dynabeads Protein A/G (Thermo Fisher Scientific) at 4 °C overnight. The Dynabeads slurry was washed four times with ice-cold wash buffer (10 mM Tris-HCl (pH 8.0), 500 mM NaCl, 1 mM CaCl2, and 0.5% NP-40) and washed twice in TE buffer (10 mM Tris-HCl (pH 8.0) and 1 mM EDTA). The samples were eluted in elution buffer (50 mM Tris-HCl (pH 8.0), 10 mM EDTA, and 1% SDS). Reverse crosslinking was performed in three steps: (1) incubation with 250 mM NaCl at 65 °C for 4 h; (2) incubation with 25 μg/mL RNase A at 37 °C for 30 min; and (3) incubation with 0.1 mg/mL proteinase K at 50 °C for 1 h. The samples were purified using a QIAquick PCR Purification Kit (Qiagen, Hilden, Germany). qPCR was performed as previously described. The primers used are listed in Table S2. The enrichment of p65 was quantified using the percentage input method.

### 4.10. Luciferase Reporter Assay

DNMT3B1 cloned into the p3XFLAG-CMV10 vector has been previously described [18]. The sequences of the human CXCL8 promoter (−149 to +7) and enhancer (+35,245 to +35,785) were amplified from genomic DNA from A549 cells using KOD -Plus- Neo (TOYOBO). The primers used are listed in Table S2. The PCR products were digested with Kpn I-HF (New England BioLabs), Sac I-HF (New England BioLabs), Hind III (New England BioLabs), and Bgl II (NIPPON genetics, Tokyo, Japan) and inserted into the similarly digested pGL4.14[luc2/Hygro] vector (Promega) using Ligation High Ver.2 (TOYOBO). All the constructed plasmids and inserted sequences were verified by sequencing. Both vectors were transiently transfected into HEK293T cells using polyethyleneimine (PEI)-max (Polysciences). Cells were treated with 1,2-NQ with medium changes at 6 h post-transfection. After 72 h, a luciferase assay was performed using the luciferase assay system (Promega) according to the manufacturer’s protocol. Luminescence intensity was measured using the Glomax Multi Detection System (Promega).

### 4.11. Assessment of Cell Proliferation

Cell proliferation was detected using a Cell Counting Kit-8 (Dojindo, Rockville, MD, USA). A549 cells were serum starved for 24 h and then incubated with SCH-527123 for 4 h before exposure to 1,2-NQ. Cell proliferation was assessed by measuring the OD450 value.

### 4.12. Clinical Data

Gene expression profiles and clinical data of lung adenocarcinoma patients were obtained from the cBioportal database (TCGA, Lung Adenocarcinoma (LUAD), Firehose Legacy) [68,69,70]. Statistical analyses were performed using GraphPad Prism version 10 (GraphPad, Boston, MA, USA).

### 4.13. p65 Nuclear Translocation Assay

Nuclear extraction was performed as described previously [71]. The cytoplasmic and nuclear fractions were quantified using the TaKaRa Bradford protein assay kit (TaKaRa) according to the manufacturer’s protocol and boiled in 1× Laemmli SDS sample buffer (62.5 mM Tris–HCl (pH 6.8), 5% 2-mercaptoethanol, 2% SDS, and 10% glycerol) for 5 min. The samples were separated using sodium dodecyl sulfate–polyacrylamide gel electrophoresis and transferred to polyvinylidene difluoride (PVDF) membranes. The PVDF membranes were blocked with 5% nonfat dry milk in Tris-buffered saline containing 0.1% Tween-20 (TBS-T) for 1 h at RT and incubated with anti-NF-κB p65 (1:5000), anti-Lamin A/C (1:5000), and anti-α-tubulin (1:10,000) at 4 °C overnight. The PVDF membranes were washed five times with TBS-T and incubated with horseradish peroxidase (HRP)-conjugated secondary antibodies (1:25,000). The PVDF membranes were again washed five times with TBS-T, and the antibody-reactive bands were visualized via ImmunoStar LD (FUJIFILM Wako Pure Chemical Corporation) and detected using a ChemiDoc XRS+ System (Bio-Rad, Hercules, CA, USA). The blot intensity was quantified as previously described [72]. Original blots were provided Supplementary Materials (Figure S7).

### 4.14. Statistical Analysis

The data are expressed as the means ± standard errors of the mean. All statistical analyses were performed using GraphPad Prism version 10 (GraphPad). The significance of differences was analyzed using unpaired two-tailed Student’s *t* test, one-way ANOVA with Dunnett’s multiple comparisons test, Tukey’s multiple comparisons test, or Fisher’s exact test, as appropriate. Survival curves were compared using the log-rank test. Statistical significance was set at *p* < 0.05.

## Data Availability

The RNA sequencing data presented in the study are openly available in DDBJ at https://ddbj.nig.ac.jp/search/entry/sra-submission/DRA019135 (accessed on 30 August 2024).

## Supplementary Materials

The following supporting information can be downloaded at: https://www.mdpi.com/article/10.3390/ijms252111592/s1, References [73,74] are cited in the Supplementary Materials.
