# Supplementary material for: Epigenetic Regulation of CXC Chemokine Expression by Environmental Electrophiles Through DNA Methyltransferase Inhibition
Source: Int J Mol Sci. 2024 Oct 29;25(21):11592. doi: 10.3390/ijms252111592 (PMC11546359; doi:10.3390/ijms252111592)
Supplement: Supplementary file 1 [file ijms-25-11592-s001.zip › Supplementary_Materials_Tsuchida_IJMS_Final.pdf]

Supplementary Materials

Article

Epigenetic Regulation of CXC Chemokine Expression by Environmental Electrophiles Through DNA Methyltransferase Inhibition

Tomoki Tsuchida [1], Sho Kubota [1], Shizuki Kamiuezono [2], Nobumasa Takasugi [1], Akihiro Ito [3], Yoshito Kumagai [4] and Takashi Uehara [1,*]

[1] Department of Medicinal Pharmacology, Graduate School of Medicine, Dentistry and Pharmaceutical
Sciences, Okayama University, Okayama 700-8530, Japan; p77b19n9@s.okayama-u.ac.jp (T.T.); sho-kubota@okayama-u.ac.jp (S.K.); ntakasu@okayama-u.ac.jp (N.T.)
[2] Department of Medicinal Pharmacology, Faculty of Pharmaceutical Sciences, Okayama University,
Okayama 700-8530, Japan; paie95d9@s.okayama-u.ac.jp
[3] School of Life Sciences, Tokyo University of Pharmacy and Life Sciences, Tokyo 192-0392, Japan; aito@toyaku.ac.jp
[4] Graduate School of Pharmaceutical Sciences, Kyushu University, Fukuoka 812-8582, Japan; kumagai.yoshito.864@m.kyushu-u.ac.jp
* Correspondence: uehara-t@okayama-u.ac.jp; Tel.: +81-86-251-7939

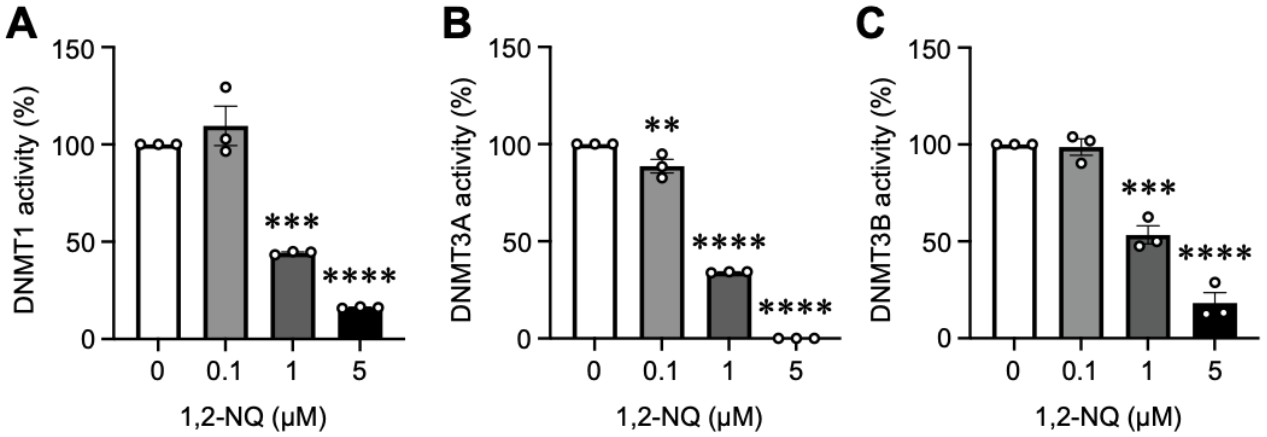

Supplementary Figure S1 1,2-NQ inhibits all DNMT subtypes.

(**A-C**) Effects of the indicated concentrations of 1,2-NQ on (**A**) DNMT1, (**B**) DNMT3A, and (**C**) DNMT3B activities. The data are expressed as the means ± SEMs. *n*=3, ***p*<0.01, ****p*<0.001, *****p*< 0.0001 versus 0 μM 1,2-NQ. Statistical analyses were performed via one-way ANOVA with Dunnett's multiple comparison test. 1,2-NQ; 1,2-naphthoquinone

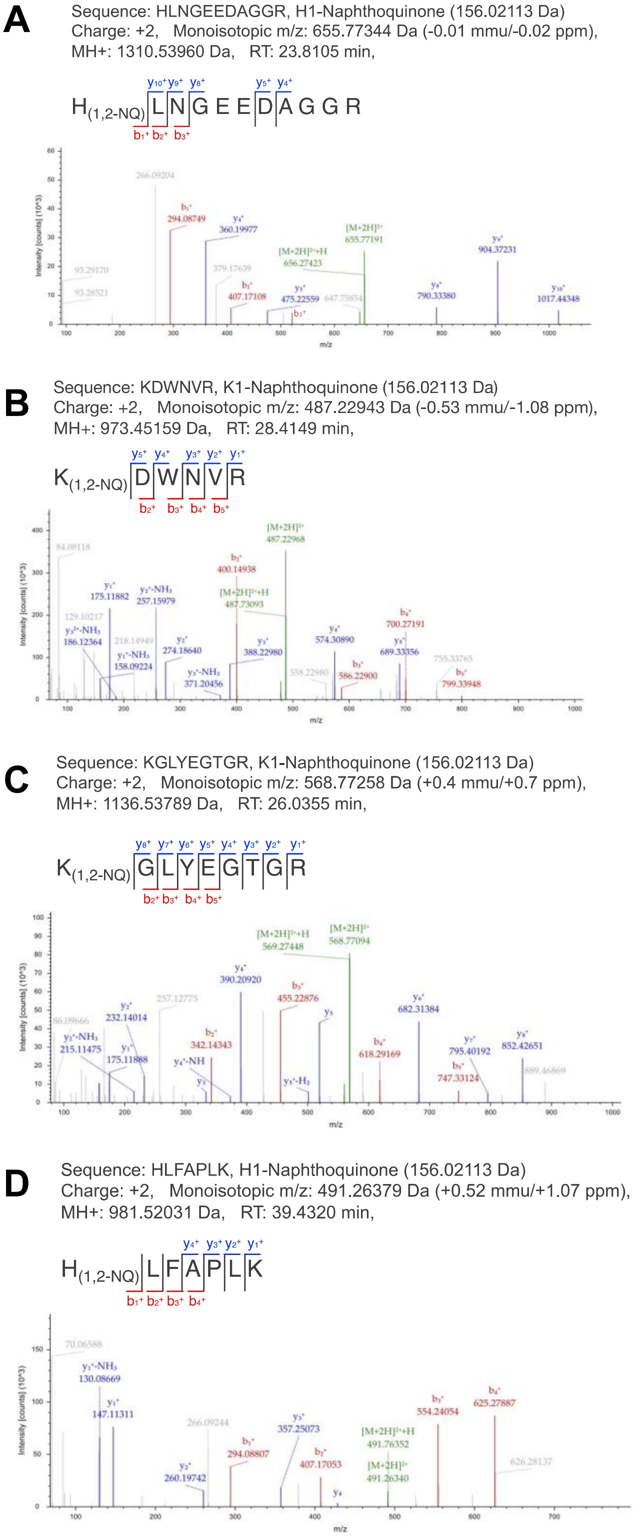

Supplementary Figure S2 Identification of 1,2-NQ modification sites in DNMT3B

(**A-D**) LC–MS/MS spectra of (**A**) H7-, (**B**) K540-, (**C**) K662-, and (**D**) H841-containing peptides. 1,2-NQ; 1,2-naphthoquinone

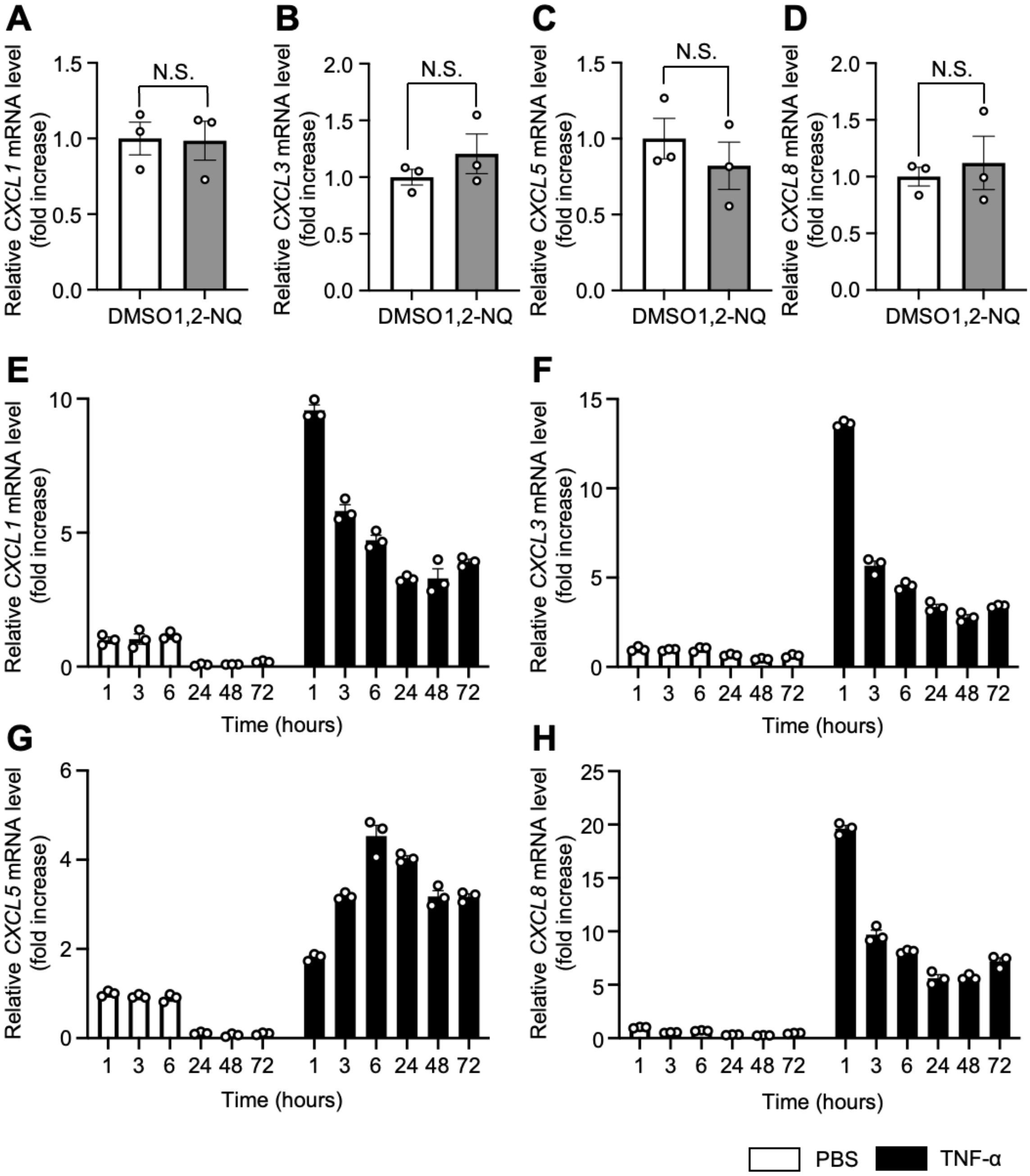

Supplementary Figure S3 1,2-NQ and TNF-α induce CXC chemokine expression via different mechanisms.

(**A-D**) A549 cells were exposed to 10 μM 1,2-NQ for 24 h, after which total RNA was extracted. RT–qPCR analysis of (**A**) *CXCL1*, (**B**) *CXCL3*, (**C**) *CXCL5*, and (**D**) *CXCL8* expression. Control DMSO is white, and 1,2-NQ is gray. The data are expressed as the means ± SEMs. *n*=3; N.S.: not significant. Statistical analyses were performed using unpaired two-tailed Student's t tests.

(**E-H**) A549 cells were exposed to 25 ng/mL TNF-α for the indicated times, and total RNA was extracted. RT–qPCR analysis of (**E**) *CXCL1*, (**F**) *CXCL3*, (**G**) *CXCL5*, and (**H**) *CXCL8* expression. The control group (PBS) is shown in white, and the TNF-α group is shown in black. The data are expressed as the means ± SEMs. *n*=3.

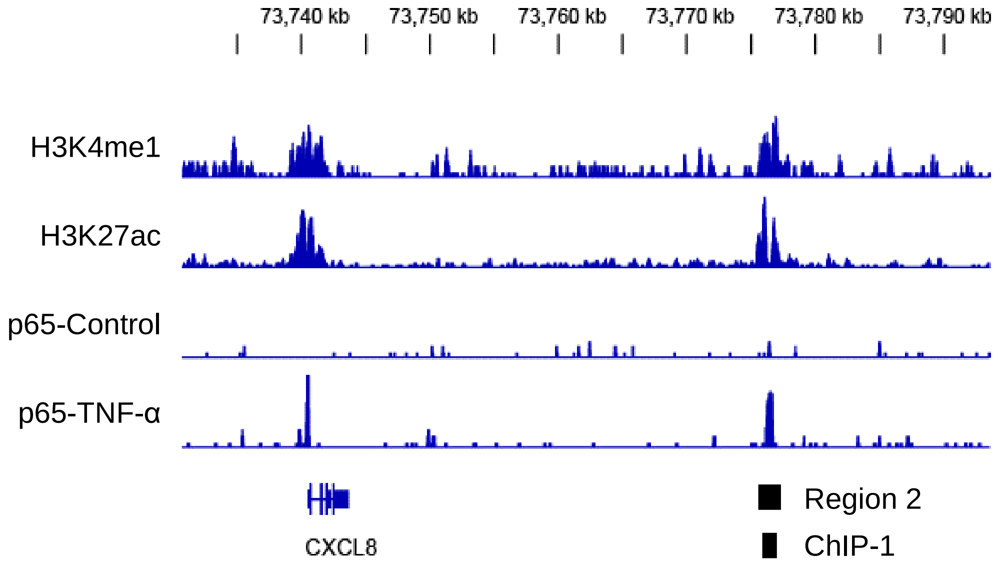

Supplementary Figure S4 Designation of the target regions in bisulfite sequencing and ChIP-qPCR

An IGV genome viewer screenshot of the ChIP-seq datasets obtained from NCBI GEO (https://www.ncbi.nlm.nih.gov/geo/). The datasets of H3K4me1 (GSM2746536), H3K27ac (GSM2746538), p65-Control (GSM2746541) and p65-TNF-α (GSM2746542) were previously reported [1,2].

References

1. Rahnamoun, H.; Lu, H.; Duttke, S.H.; Benner, C.; Glass, C.K.; Lauberth, S.M. Mutant p53 shapes the enhancer landscape of cancer cells in response to chronic immune signaling. *Nat Commun* **2017**, *8*, 754, doi:10.1038/s41467-017-01117-y.
2. Rahnamoun, H.; Lee, J.; Sun, Z.; Lu, H.; Ramsey, K.M.; Komives, E.A.; Lauberth, S.M. RNAs interact with BRD4 to promote enhanced chromatin engagement and transcription activation. *Nat Struct Mol Biol* **2018**, *25*, 687-697, doi:10.1038/s41594-018-0102-0.

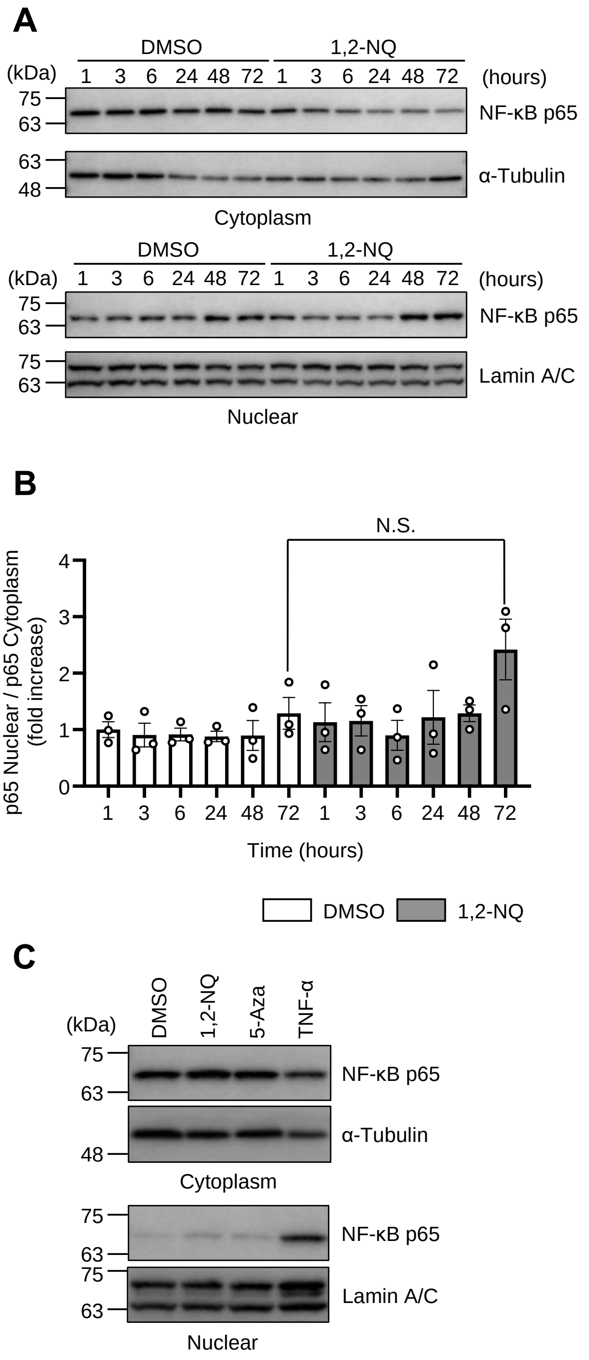

Supplementary Figure S5 1,2-NQ did not induce NF-κB p65 nuclear translocation.

(**A, B**) A549 cells were exposed to 10 μM 1,2-NQ for 72 h, (**C**) A549 cells were exposed to 10 μM 1,2-NQ, 10 μM 5-Aza, or 25 ng/mL TNF-α for 3 h, and the nuclear and cytoplasmic fractions were extracted. The samples were analyzed via western blotting with anti-p65, anti-α-tubulin (cytoplasmic marker), and anti-lamin A/C (nuclear marker) antibodies. The levels of p65 nuclear and cytoplasmic proteins were quantified and normalized to those of α-tubulin and Lamin A/C, respectively. The control (DMSO) group is shown in white, and the 1,2-NQ group is shown in gray. The data are expressed as the means ± SEMs. *n*=3; NS, not significant. Statistical analyses were performed via one-way ANOVA with Tukey's multiple comparison test.

A

Subtype	Sequence												
DNMT1	97-	H	A	Y	N	R	E	V	N	G	R	-	-106
DNMT3A	18-	E	R	E	E	D	R	K	D	G	E	E	-28
DNMT3B	7-	-	-	-	-	-	H	L	N	G	E	E	-12

B

Subtype	Sequence												
DNMT1	726-	Q	N	K	N	R	I	S	W	V	G	E	-736
DNMT3A	598-	-	-	-	-	R	E	D	W	P	S	R	-604
DNMT3B	539-	-	-	-	-	R	K	D	W	N	V	R	-545

C

Subtype	Sequence												
DNMT1	1232-	M	N	R	F	N	S	R	T	Y	S	K	-1242
DNMT3A	716-	V	N	P	A	R	K	G	L	Y	E	G	-726
DNMT3B	657-	V	N	P	A	R	K	G	L	Y	E	G	-667

D

Subtype	Sequence												
DNMT1	1462-	R	K	N	G	R	S	S	S	G	A	L	-1472
DNMT3A	900-	-	-	-	-	-	H	L	F	A	P	L	-905
DNMT3B	841-	-	-	-	-	-	H	L	F	A	P	L	-846

Supplementary Figure S6 Amino acid sequence alignment of DNMT family members for the sequence around the 1,2-NQ modification sites

(**A-D**) Amino acid sequence alignment around (**A**) H7, (**B**) K540, (**C**) K662, and (**D**) H841 in all the DNMT subtypes. The 1,2-NQ modification sites are shown in red. Gray indicates identical amino acids. 1,2-NQ; 1,2-naphthoquinone

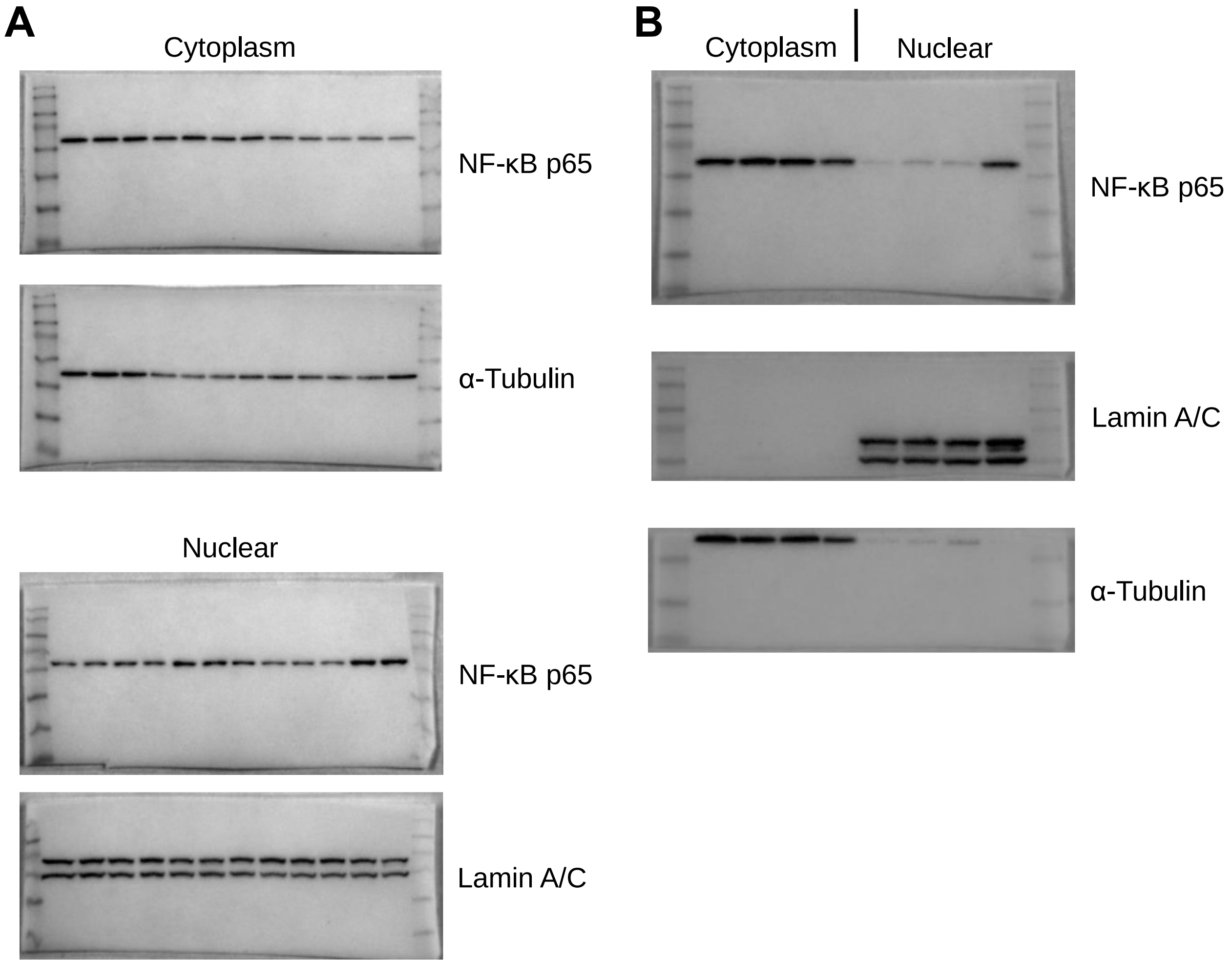

Supplementary Figure S7 Original images for western blots.

(**A**) Original blots of figure S5A. (**B**) Original blots of figure S5C.
